# A *de novo* meningioma with rapid growth: A possible malignancy imposter?

**DOI:** 10.1515/biol-2025-1118

**Published:** 2025-06-12

**Authors:** Zhenjiang Pan, Jing Bao, Shepeng Wei

**Affiliations:** Department of Neurosurgery, Shidong Hospital, Shanghai, 200438, China; Department of Neurosurgery, Shidong Hospital, No. 999, Shiguang Road, Yangpu District, Shanghai, 200438, China

**Keywords:** *de novo*, meningioma, central nervous system, surgical treatment, fibrous meningioma

## Abstract

Meningiomas, accounting for approximately 33% of primary central nervous system tumors, are the most prevalent type in this category. Advanced age is frequently viewed as a barrier to surgical intervention, yet recent cases have challenged this perception by demonstrating successful outcomes in elderly patients. This case report aims to illustrate the feasibility and benefits of surgical treatment in older individuals. An 84-year-old patient presented with a newly diagnosed meningioma and underwent surgical tumor removal at age 88. Following a comprehensive preoperative evaluation that excluded significant comorbidities, the procedure utilized advanced surgical techniques and optimized postoperative care to ensure safety and recovery. Pathology revealed a World Health Organization grade 1 fibrous meningioma, confirming its benign nature. The patient tolerated the surgery well and recovered successfully, marking her as the oldest reported individual to undergo such treatment. This case demonstrates that advanced age does not inherently limit tumor growth or preclude surgical intervention. Through meticulous patient assessment and personalized treatment strategies, elderly patients can achieve positive outcomes. It highlights the value of a tailored approach, prioritizing overall health and specific medical needs, and supports proactive surgical management to enhance quality of life and clinical results. This challenges traditional assumptions about age-related restrictions on surgical feasibility.

## 1 Introduction

Meningiomas are the most common primary tumors of the central nervous system (CNS), accounting for approximately one-third of all primary brain and spinal cord tumors [1]. With a median diagnostic age of 65 years, their incidence rises significantly with advancing age [2]. Notably, these tumors are two to three times more prevalent in women than in men, underscoring a striking gender disparity [3,4].

The World Health Organization (WHO) classifies the majority of meningiomas as grade 1, characterized by benign and slow-growing behavior. However, grades 2 (atypical) and 3 (anaplastic) meningiomas, though less common, exhibit aggressive features and higher recurrence rates, posing substantial clinical challenges [5]. Intriguingly, hospital-based studies, especially from tertiary care centers, reveal a disproportionately higher prevalence of these aggressive grades compared to general population data – likely reflecting referral biases and the complexity of cases treated at such institutions [6,7].

Thanks to advancements in neuroimaging and molecular pathology, our understanding of meningioma etiology has deepened, particularly regarding genetic and environmental factors. These insights are paving the way for targeted therapeutic strategies aimed at higher-grade meningiomas [8].

In this context, we report a compelling case of an 84-year-old patient who developed a new meningioma, which was surgically removed at the age of 88. This patient is likely the oldest individual with a WHO grade 1 fibrous meningioma to have undergone successful surgical treatment to date. Her case underscores the vital role of individualized treatment strategies for elderly patients, demonstrating that advanced age alone should not preclude surgical intervention. The patient’s remarkable outcome aligns with emerging evidence advocating for tailored approaches to maximize benefits, even in the aging population [9,10].

## 2 Case report

**Figure 1:**
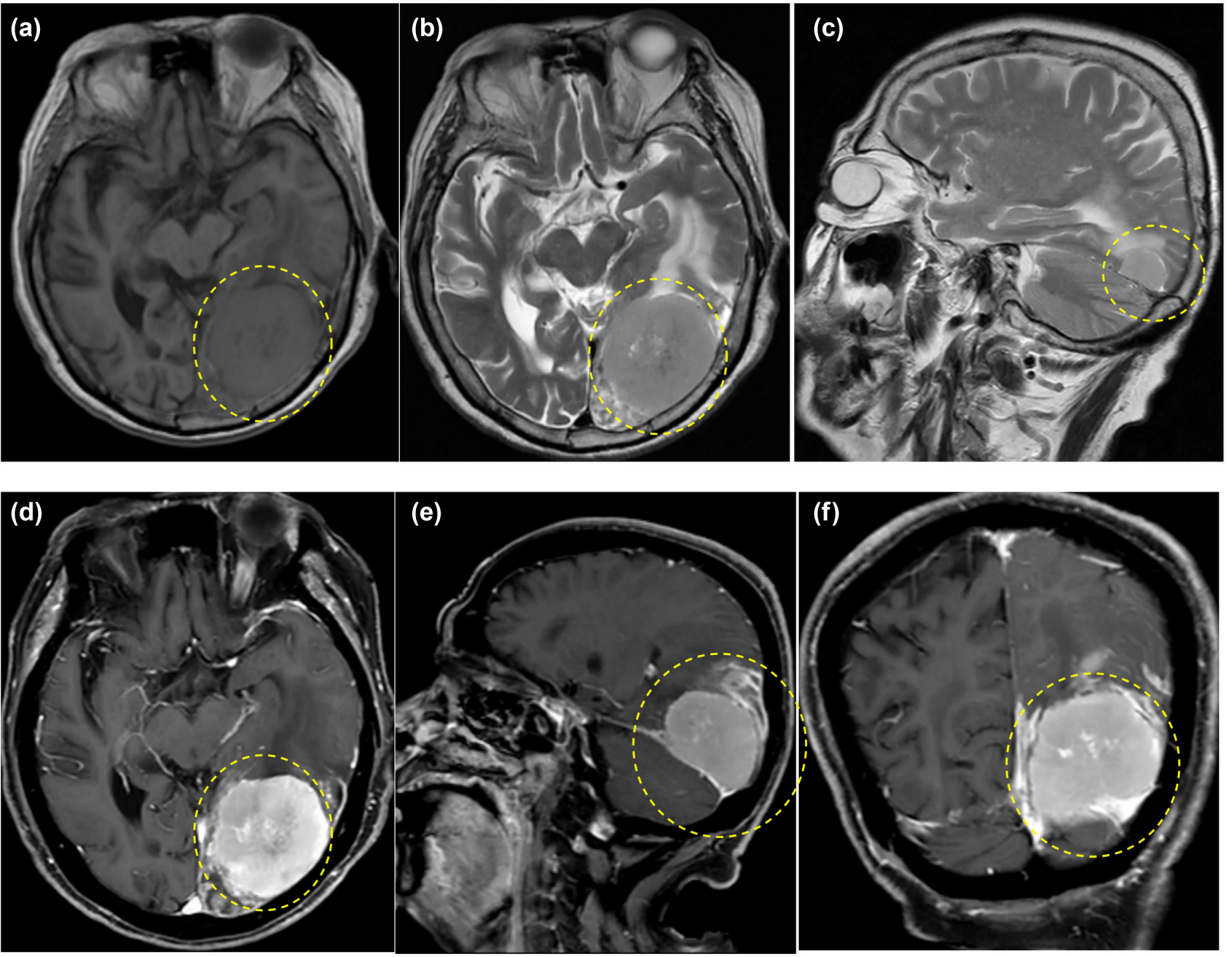
Brain MRI images demonstrating a large left-sided parasagittal occipital extra-axial mass with heterogeneous enhancement, with the base of the tumor located on the tentorium cerebelli (yellow dashed circle). The mass is isointense to grey matter on T1-weighted images and hyperintense on T2-weighted images (a)–(c); the calcified components are hypointense on T2-weighted images. There is marked contrast enhancement on T1-weighted images with gadolinium (d)–(f).

**Figure 2:**
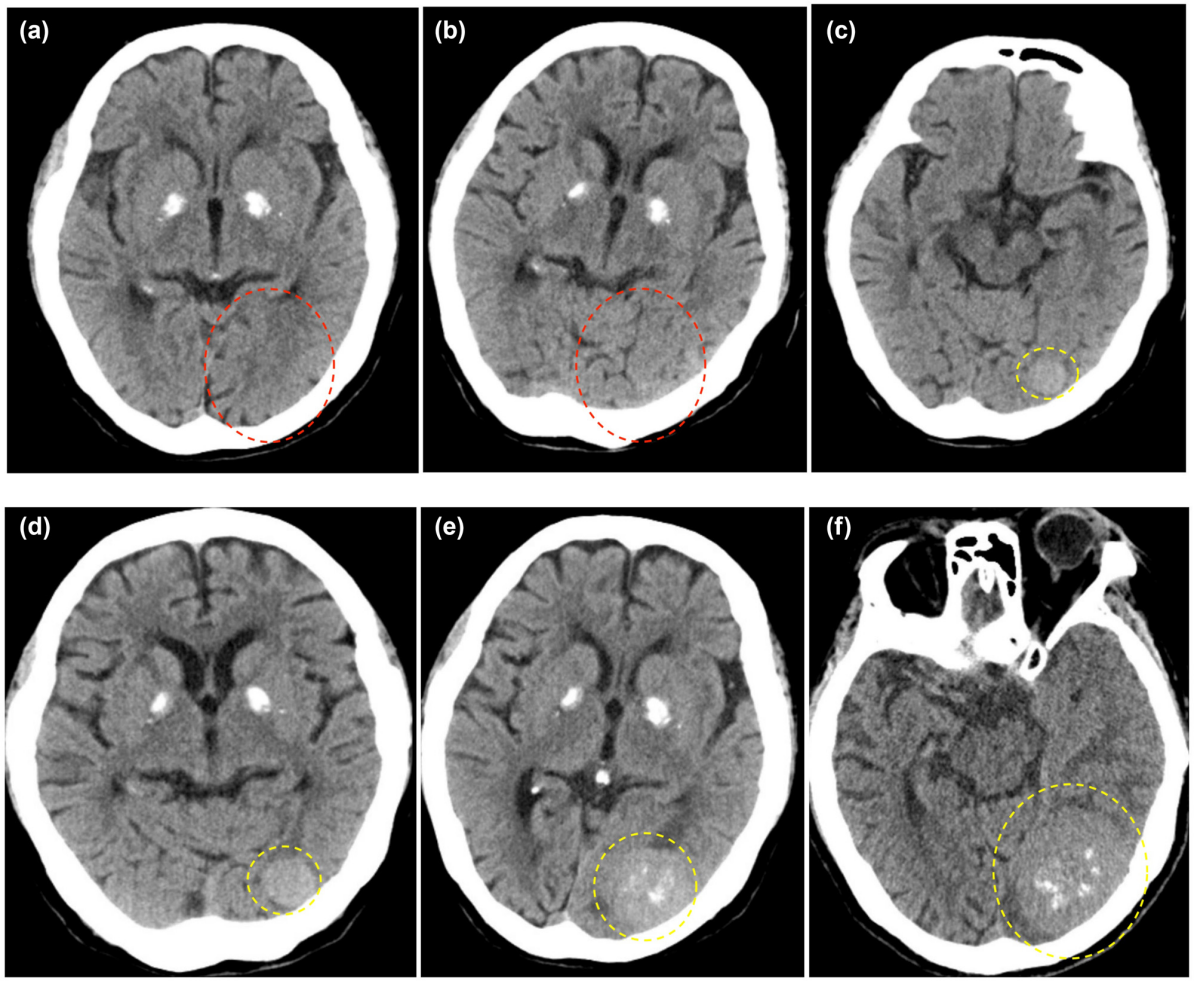
A series of axial CT scans from 2015 to 2023 shows no tumor within the red dashed circle. Within the yellow dashed circle, a tumor is observed growing progressively larger over the years. The mass, located in the occipital region close to the tentorium cerebelli, appears isodense with scattered areas of calcification in its center. The scans correspond to the years 2015 (a), 2018 (b), 2019 (c), 2020 (d), 2021 (e), and 2023 (f).

**Figure 3:**
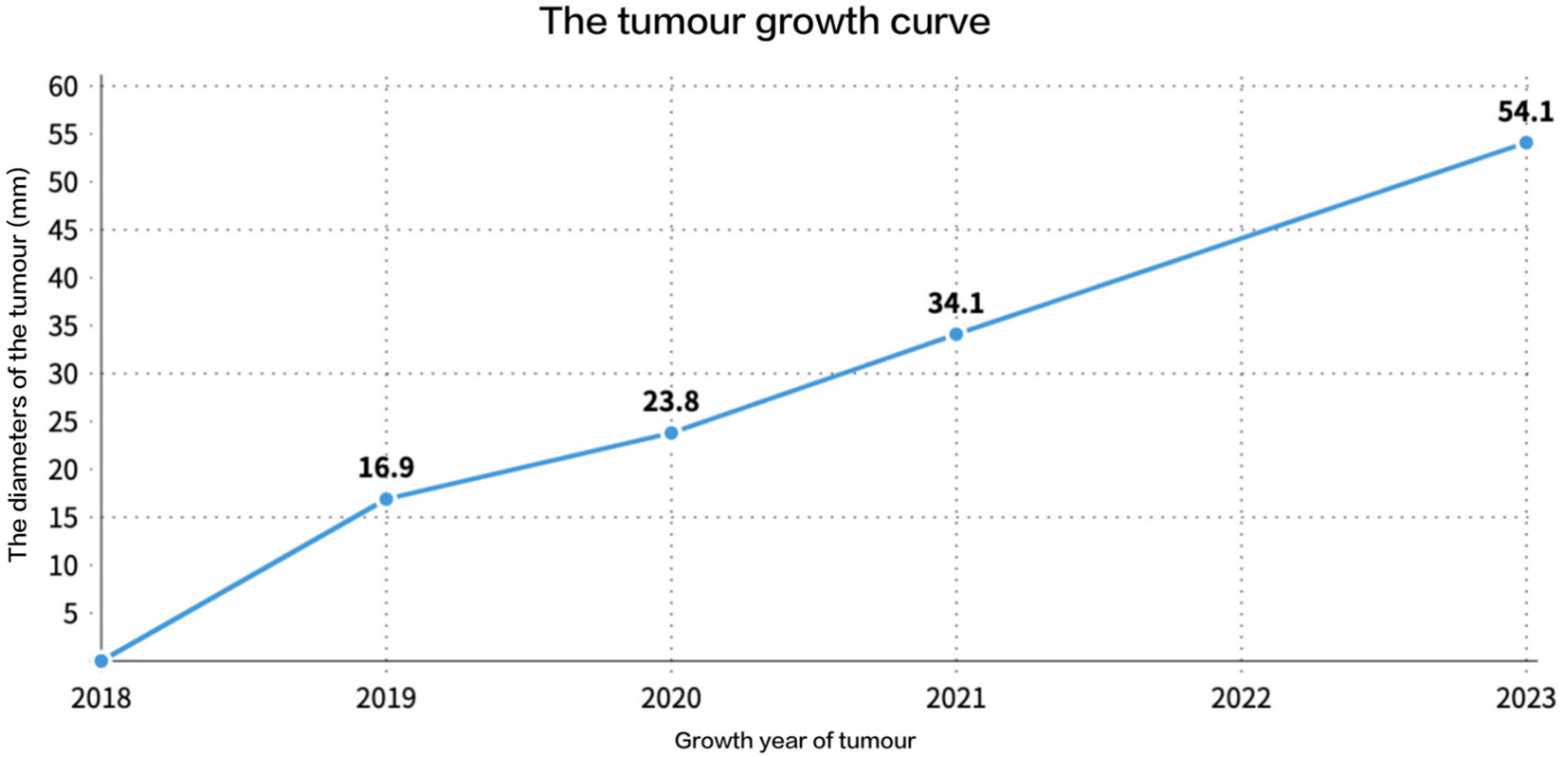
The tumor growth curve illustrates an increase in diameter from 16.9 mm in 2019 to 54.1 mm in 2023. The annual growth rate of the tumor diameter was approximately 9 mm/year before surgery.

**Figure 4:**
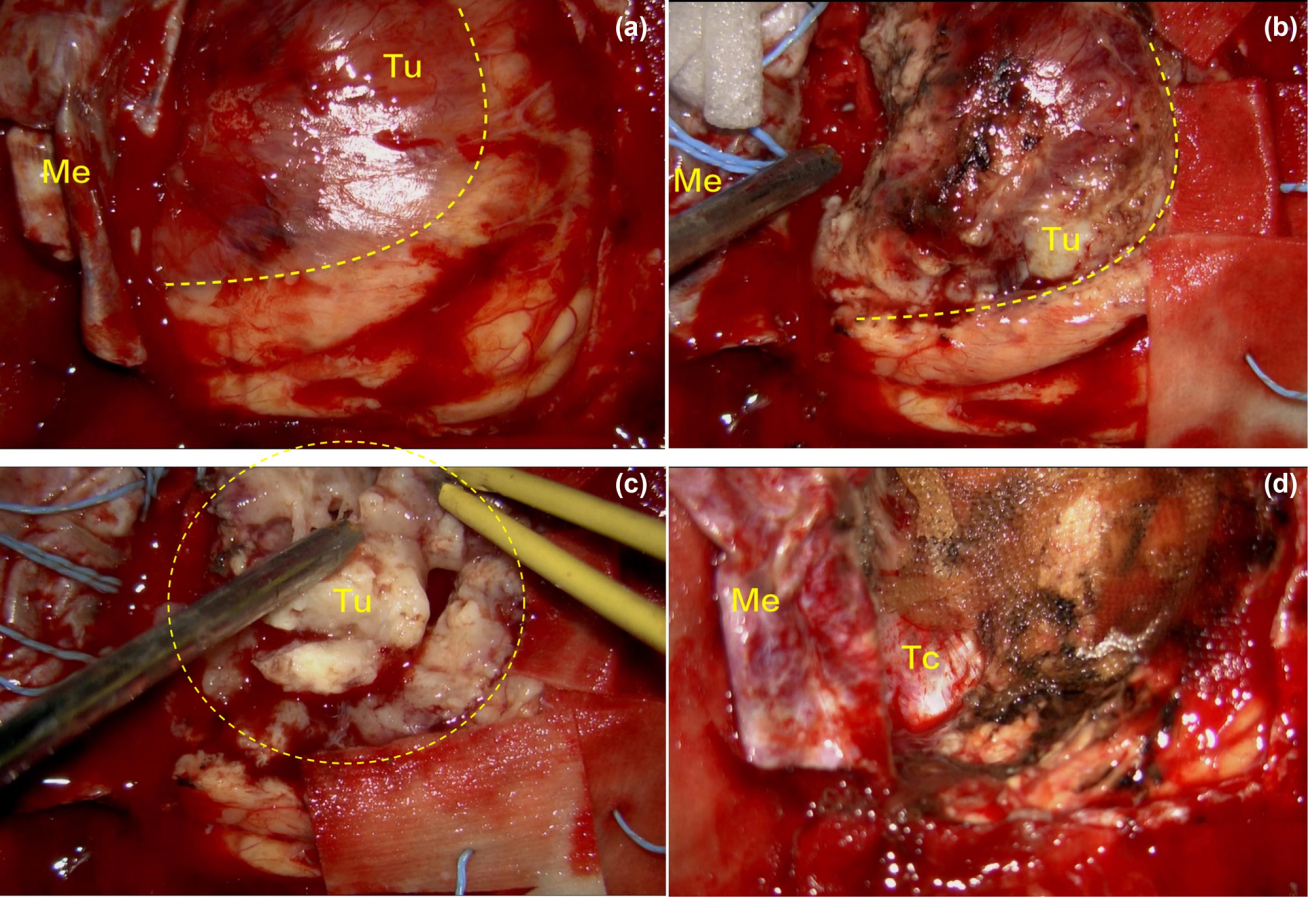
Photographs were obtained through an operating microscope from the surgeon’s perspective. The areas within the yellow dashed lines and circles represent the tumor. In panel (a), immediately after the dura mater (Me) was opened, the dividing line (yellow dashed line) between the tumor (Tu) and brain tissue is visible. Following the opening of the arachnoid membrane, panel (b) displays the tumor (Tu). Panel (c), taken after partial resection, reveals that the tumor’s texture was slightly soft and its blood supply was moderate. Panel (d) shows that the tentorium cerebelli (Tc) is fully visible after the tumor was completely resected.

**Figure 5:**
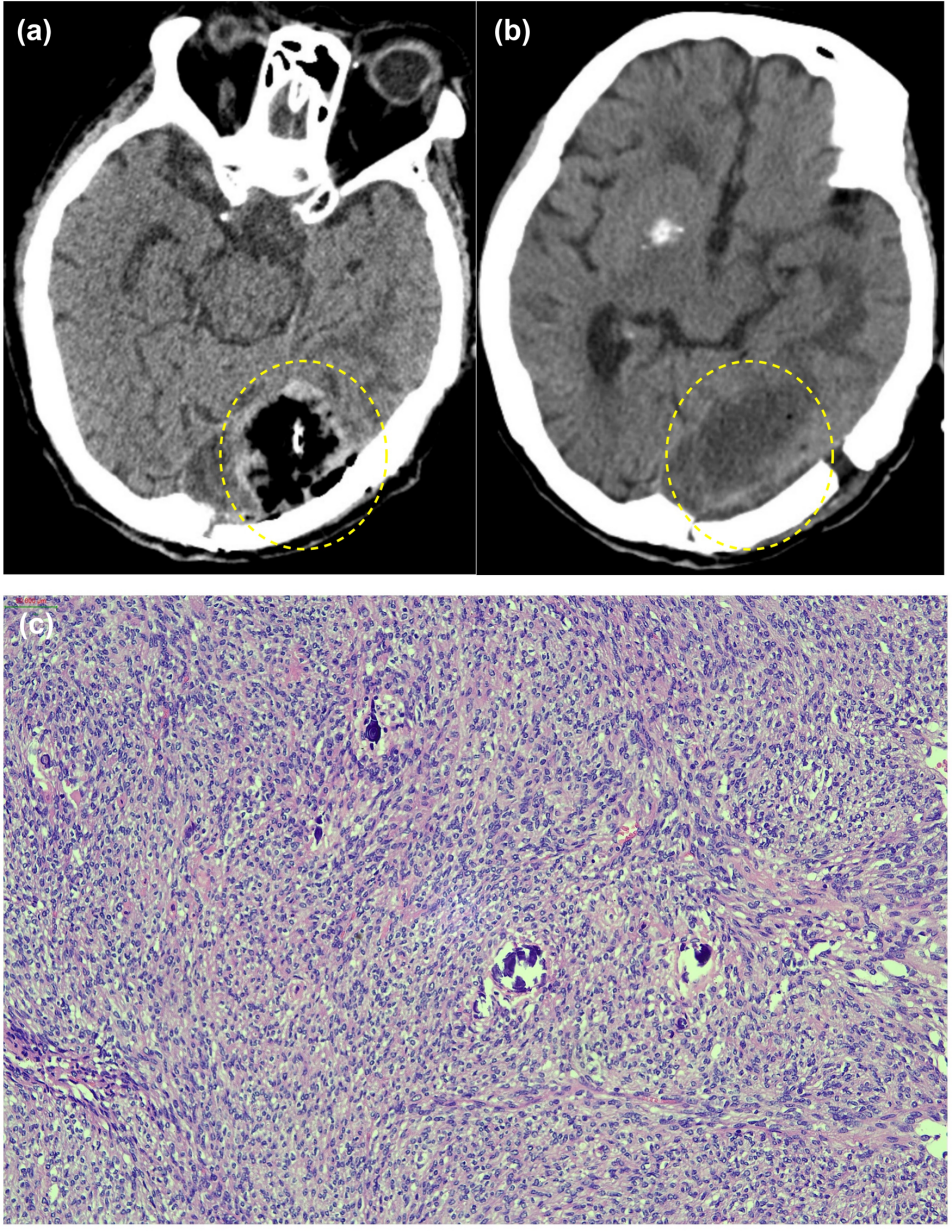
Postoperative pathology and imaging. No residual tumor was observed within the yellow dashed circle. Panel (a) displays the head CT on the second day postoperatively, while panel (b) displays it on the eighth day. The operation field is clean, and there is no intracranial bleeding. Panel (c) presents the pathology of a Grade 1 fibrous meningioma, with a Ki-67 index of 1%.


An 88-year-old female (weight: 65 kg, BMI: 23.5) presented with a 2-month history of dizziness, blurred vision, and progressive memory decline. Her medical history included type II diabetes mellitus, hypertension, and a 5-year history of anxiety disorder managed with fluoxetine (20 mg daily), with no known drug allergies. Neurological examination revealed normal speech and intact muscle strength (Grade 5) across all major muscle groups; however, visual acuity and fields could not be assessed due to poor cooperation. Brain MRI disclosed a large, heterogeneously enhancing left parasagittal occipital extra-axial mass anchored to the tentorium cerebelli (Figure 1). Retrospective imaging review showed no tumor on CT scans from 2015 to 2018, with the lesion first detected in December 2019 (16.9 mm), exhibiting slow growth to 34.1 mm by September 2021 and accelerated expansion to 54.0 mm by 2023, spanning ages 84–88 (Figures 2 and 3). Under general anesthesia with three-pin head fixation in the lateral prone position, a horseshoe-shaped incision oriented toward the transverse sinus was made. Microscopic dural opening revealed a grayish-white tumor with well-defined margins, no adhesions to the falx cerebri, and moderate vascularity (Figure 4). Originating from the tentorium cerebelli, the tumor was resected *en bloc* within 2 h, achieving a Simpson Grade 2 resection with local cauterization of the tentorial base. Intraoperative parameters remained stable, with an estimated blood loss of 200 mL and no transfusion required. Post-anesthesia assessment confirmed the patient was conscious, with slow but accurate responses and purposeful limb movements. The postoperative course was unremarkable, with no fever observed. Sutures were removed on postoperative Day 8, and the incision healed excellently (Grade I/A). Postoperative CT scans showed no hemorrhage on Day 2 and occipital lobe rebound by Day 9. Pathology confirmed a WHO Grade 1 fibrous meningioma (Figure 5). The patient was discharged on postoperative Day 11, alert, with fluent speech and preserved neurological function (Grade 5 strength). A telephone follow-up in November 2023, 6 months post-surgery, confirmed her survival and ability to live independently in a nursing home.

**Informed consent:** Informed consent has been obtained from all individuals included in this study.
**Ethical approval:** The research related to human use has been complied with all the relevant national regulations, institutional policies and in accordance with the tenets of the Helsinki Declaration, and has been approved by the authors’ institutional review board or equivalent committee.

## 3 Discussion

### 3.1 A remarkable case of rapidly growing meningioma in an 88-year-old woman

This case tells the compelling story of an 88-year-old woman diagnosed with a newly developed meningioma. Over just 3 years, the tumor grew dramatically from 16 to 54.1 mm in diameter, defying expectations typically associated with advanced age. Initially discovered at age 84, the meningioma measured a mere 16.9 mm, with no signs of brain damage – a finding that justified adopting a conservative “watch and wait” approach, often recommended for small tumors in older patients with limited life expectancy or comorbidities [11].

By the time the patient reached 86, the tumor had grown to 34.5 mm. Despite the significant increase in size, there were no clinical signs of increased intracranial pressure or peritumoral edema (Figure 1e), and the patient remained asymptomatic. Regular follow-up was maintained, aligning with findings from a meta-analysis of 2,130 patients showing that 51% of those with incidental, asymptomatic meningiomas underwent active monitoring, and only 25% required intervention over an average follow-up period of 4 years [12].

However, the story took an unexpected turn. Over the next 2 years, the tumor’s growth accelerated, averaging 11 mm annually, and reached 55.5 mm by the time the patient was 88. Alarmingly, the presence of surrounding edema suggested the possibility of a higher-grade meningioma, raising concerns about the patient’s prognosis [13–15]. Surprisingly, the pathological analysis post-surgery revealed a WHO grade 1 fibrous meningioma (Figure 3c), reaffirming its benign nature. This finding stands in contrast to recent reviews suggesting a higher prevalence of grade 2 meningiomas in patients aged 80 and older [16].

Despite the tumor’s rapid progression, the patient’s lack of significant comorbidities allowed for a craniotomy, which successfully removed the mass. The operation proceeded without complications, and the patient made a smooth recovery, returning home shortly thereafter. This case demonstrates that even in the context of rapid tumor growth and advanced age, surgical intervention remains a viable and effective option.

## 4 Challenging preconceptions: Surgery in the elderly

This case serves as a powerful reminder that advanced age neither slows tumor growth nor inherently contraindicates surgical treatment. While the patient’s tumor exhibited rapid progression, timely intervention, guided by careful monitoring, led to a successful outcome. Emerging evidence suggests that neurosurgical procedures in appropriately selected elderly patients can yield outcomes comparable to those of younger cohorts, provided there are no significant comorbidities [15,16].

Maiuri et al. investigated intracranial meningiomas in patients aged ≥80 years, highlighting their pathological features and surgical challenges. Their study underscores that surgery remains feasible even in very elderly patients, with a notable case involving a 96-year-old individual – surpassing the age of my 88-year-old patient. Key findings emphasize that tumor characteristics, rather than age alone, should guide treatment decisions. Surgical risks, though present, can be mitigated with careful patient selection and tailored approaches. This evidence challenges age-related contraindications, supporting individualized management [16]. My patient’s case aligns with this, though their age is not the upper limit observed.

The findings from this case underscore the importance of personalized treatment plans that consider the patient’s overall health, tumor characteristics, and the potential for improved quality of life. They challenge the long-held notion that advanced age precludes surgery, advocating instead for proactive decision-making tailored to individual circumstances. By embracing this approach, clinicians can help elderly patients achieve outcomes that enhance both their quality of life and clinical prognosis, even when managing benign CNS tumors.

## 5 Conclusion

Meningiomas, among the most common primary brain tumors, have a striking prevalence in the elderly population. According to the WHO, approximately 80–85% of these tumors are classified as grade 1, underscoring their typically benign nature. Yet, their impact on patients’ quality of life, particularly in advanced age, poses critical challenges.

Thanks to remarkable advancements in science and technology, the landscape of meningioma management has undergone a transformative shift. Our findings illuminate a key paradigm: advanced age should no longer be viewed as a contraindication for surgical intervention. On the contrary, for elderly patients without significant comorbidities, surgical removal of meningiomas is not only feasible but also often life-enhancing.

By prioritizing tailored, patient-specific approaches, modern neurosurgical techniques empower clinicians to achieve successful outcomes, even in older adults. These results challenge outdated assumptions and affirm that age alone is not a barrier to effective treatment. With proper evaluation and intervention, elderly patients can enjoy a restored quality of life, exemplifying the power of precision medicine and innovative care.
